# Gut Fungal Microbiome Responses to Natural *Cryptosporidium* Infection in Horses

**DOI:** 10.3389/fmicb.2022.877280

**Published:** 2022-07-06

**Authors:** Yaping Wang, Xuwen Li, Xiushuang Chen, Muhammad Fakhar-e-Alam Kulyar, Kun Duan, Huade Li, Zeeshan Ahmad Bhutta, Yi Wu, Kun Li

**Affiliations:** ^1^Institute of Traditional Chinese Veterinary Medicine, College of Veterinary Medicine, Nanjing Agricultural University, Nanjing, China; ^2^MOE Joint International Research Laboratory of Animal Health and Food Safety, College of Veterinary Medicine, Nanjing Agricultural University, Nanjing, China; ^3^College of Veterinary Medicine, Huazhong Agricultural University, Wuhan, China; ^4^China Tobacco Henan Industrial Co., Ltd., Zhengzhou, China; ^5^Sichuan Academy of Grassland Science, Chengdu, China; ^6^Laboratory of Biochemistry and Immunology, College of Veterinary Medicine, Chungbuk National University, Cheongju, South Korea

**Keywords:** *Cryptosporidium* infection, prevalence, fungi microbiota, horse, protozoa

## Abstract

It is critical to characterize changes in the structure and composition of the host fungal community in natural *Cryptosporidium* infection, because it gives the possible overview of gut microbiome in host homeostasis and disease progression. A total of 168 rectal fecal samples were collected and examined using nPCR. The positive samples were double-checked using 18S rDNA high-throughput sequencing. After confirmation, ITS high-throughput sequencing was utilized to investigate the fungal community’s response to natural *Cryptosporidium* infection. Results showed that a total of three positive samples (1.79%) were identified with an increased abundance of fungi associated with health hazards, such as class Dothideomycetes, families, i.e., Cladosporiaceae, Glomerellaceae, and genera, i.e., *Wickerhamomyces*, *Talaromyces*, *Cladosporium*, *Dactylonectria*, and *Colletotrichum*. On the contrary, taxa associated with favorable physiological effects on the host were shown to have the reverse impact, such as families, i.e., Psathyrellaceae, Pseudeurotiaceae and genera (*Beauveria*, *Nigrospora*, and *Diversispora*). For the first time, we evaluated the condition of natural *Cryptosporidium* infection in horses in Wuhan, China, and discovered distinct variations in the fungal microbiome in response to natural infection. It might prompt a therapy or prevention strategy to apply specific fungal microorganisms that are probably responsible for decreased susceptibility or increased resistance to infection.

## Introduction

The gut is a diverse and complex ecosystem of microbes, encompassing viruses, bacteria, fungi, parasites, and other microorganisms that have been demonstrated to play a fundamental role in maintaining the health or disease state of the host (Chin et al., 2020). The intestinal immune system has evolved to adequately meet the presence of various microorganisms, including beneficial, detrimental, and harmless microbes (Hooper et al., 2012). The host intestinal immune system has to make accurate assessments to accept or reject the new microbes in each condition. Meanwhile, the incoming organism has also evolved to maximize opportunities for acceptance (Reynolds et al., 2015). Hence, investigating the interplay between various diseases and gut microbiota remains the key focus. Evidence shows that the symbiotic relationship between host and resident microbial taxa in the gut contributes to extracting essential nutrients as a first-line for gut pathogens and shaping the immune system [Human Microbiome Project Consortium [HMPC], 2012]. There is an increasing realization that the influences between the parasites and gut microbiota are intertwined. Reynolds et al. (2015) reported that parasitic infection changed the structure and composition of the gut microbiome, and conversely, the stable microbiota affects intestinal parasite colonization and persistence in the gut. Infection with parasites can disrupt or restructure the host’s gut communities, both in vertebrates and invertebrates (Hayes et al., 2010; Charania et al., 2020). On the contrary, a disrupted intestinal microbiome can increase the susceptibility to some parasitic diseases, e.g., malaria and *Giardia* infections (Villarino et al., 2016; Barash et al., 2017).

Interaction between intestinal organisms in host individuals is one of the key determinants of dynamics in intestinal communities. These multitudes of interactions are both detrimental and beneficial to their hosts, affecting symbiont communities’ ecological niche and evolution (Pedersen and Fenton, 2007; Rigaud et al., 2010). Recently, few observational studies reported that the interactions between parasites and microbiota have become more feasible on the base of new sequencing technologies. Some have reported no bacterial community changes in infection, whereas others did (Cooper et al., 2013; Lee et al., 2021). In all conditions with significant alterations, a higher diversity of bacteria has been associated with infections. At the same time, the impact on operational taxonomic units (OTUs) have also been identified to have parasitic specificity (Aivelo and Norberg, 2018). Even though most research is focused on the shifts of gut bacterial communities in parasites infections. Growing evidence confirmed the undisputable effects of fungi in driving various gut-associated and metabolic diseases (Huseyin et al., 2017). Compared with bacterial communities, fungal presence is relatively insignificant that limits the in-depth understanding of this “rare biosphere.”

Nonetheless, deep-sequencing technologies have revealed the complexity of the fungi microflora that existed on the gut mucosal surfaces. In addition, accumulated evidence showed that specific fungi played an important role in modulating host immune response and maintaining host dynamic gut microflora changes (Iliev et al., 2012), which interacted with the host immune system through the innate immune receptor Dectin-1. More profoundly, gut fungal communities may be the reservoir for opportunistic pathogens when a host is immunocompromised (Polvi et al., 2015). For example, previous research reported that the fungal burden increased in the gut of the Crohn’s Disease patients (Ott et al., 2008). The evidence clearly indicated that fungi microflora was essential in host homeostasis and disease development, suggesting a potential role of gut fungi in host homeostasis and disease development in mammals. So, it is essential to characterize the shifts of structure and composition of host fungi community in natural parasitic infection.

*Cryptosporidium* spp. are opportunistic apicomplexan pathogenic parasites, infecting gut epithelial cells and causing debilitating gastrointestinal and diarrheal ailment in the host (Charania et al., 2020). Cryptosporidiosis can be life-threatening in immunocompromised individuals. High morbidity and mortality have been reported in patients with AIDS/HIV (Sow et al., 2016). One single *Cryptosporidium* oocyst can trigger an infection in immunosuppressed individuals (Graczyk et al., 1997). The infection can be spread directly from the infective oocysts in the feces or indirectly from vegetables or other food items (contaminated by the feces of infected hosts) (Fayer et al., 2013). The findings of *Cryptosporidium* spp. in the infected horses and donkeys, reared as companion animals suggest that these could be a reason for transmission to humans (Jian et al., 2016). In equines, most reports on cryptosporidiosis were related to Immune Deficiency Syndrome, e.g., Arabian foal (Mair et al., 1990). Moreover, it has been reported that the infection by *Cryptosporidium* in healthy foals occurred in the United States, Spain, etc. (Coleman et al., 1989). A remarkable phenomenon is the difference in the severity and susceptibility of *Cryptosporidium* infection (Gilchrist et al., 2016). These conditions may be multifactorial, including host species, immunological status, and differences in the host gut microflora.

Therefore, the study aimed to test the hypothesis that gut fungal microbiota as a holobiont could respond to natural *Cryptosporidium* infection in horses. Here, we used specific polymerase chain reaction (PCR) amplification and 18S rDNA high-throughput sequencing to reveal the occurrence of natural *Cryptosporidium* infection. In addition, we screened positively infected feces to identify changes in the structure and composition of fungi to explore any correlation to *Cryptosporidium* infection. This study was the first report that revealed natural *Cryptosporidium* infection in horses in Wuhan, China, and response of gut fungal microbiota to infection.

## Materials and Methods

### Collection of Fecal Samples

The sterile fecal collection tubes were used to collect 168 rectal fecal samples from horses in Wuhan, China. Each fecal sample (about 2 g) obtained was immediately transferred into a fecal collection tube and rapidly frozen in dry ice before storing at −80°C in the clinical lab of Huazhong Agricultural University. The study procedures were approved by the Ethics Committee of Huazhong Agricultural University and Animals Research Centre of Hubei Province.

### DNA Extraction

Before DNA extraction, samples were vortexed to homogenization. The genomic DNA (gDNA) was extracted from feces using the commercially available product E.Z.N.A^®^ Stool DNA Kit (Omega Bio-tek, Inc., United States) following the manufacturer’s recommended procedure. Then, the quality and purity of gDNA were checked using spectrophotometry and 1% agarose gel electrophoresis before storage at –20°C.

### PCR Amplification and DNA Agarose Gel Electrophoresis

Following the reports described previously, we amplified the 18S rDNA gene of *Cryptosporidium* using two-step nested PCR (Ryan et al., 2003). In the primary PCR, a pair of primers 18SiCF2: 5′-GACATATCATTCAAGTTTCTGACC-3′ and 18SiCR2: 5′-CTGAAGGAGTAAGGAACAACC-3′ were used to obtain a PCR product of 763 bp. Total 25 μl PCR reaction mixture contained 1 μl Taq, total 2 μl forward and reverse primers, 2.5 μl PCR Buffer (10×), 2.5 μl DNA, 5 μl dNTPs (2.5 mM), and 12 μl sterile distilled water. The annealing temperature was 58°C for 30 s during 45 PCR cycles. During the second PCR, a fragment of 587 bp was amplified using forward primer 18SiCF1 (5′-CCTATCAGCTTTAGACGGTAGG-3′) and reverse primer 18SiCR1 (5′-TCTAAGAATTTCACCTCTGACTG-3′). The total 50 μl reaction volume contained a total of 4 μl forward and reverse primers, 2 μl Taq, 5 μl primary PCR product, 5 μl PCR Buffer (10 × ), 10 μl dNTPs (2.5 mM), and 24 μl sterile distilled water. The conditions for two-step PCR were identical. Ultimately, all PCR products were analyzed using 1% agarose gels (w/v).

### Double-Test to Verify *Cryptosporidium* Spp. Infection Using 18S rDNA High-Throughput Sequencing

The *Cryptosporidium*-positive feces verified by the nested PCR were double-checked to confirm infection using 18S rDNA high-throughput sequencing. Specific primers based on the V4 region were synthesized for amplification (F: 5′-CCAGCASCYGCGGTAATTCC-3′, and R: 5′-ACTTTCGTTCTTGATYRA-3′). The annealing temperature was 56°C for 30 s during 35 PCR cycles. All the PCR amplification products were performed 1% agarose gel electrophoresis, then the gel recovery kit (Axygen, United States) was used to recover targeted fragments. Subsequently, purified all PCR products to remove the unspecific products for library preparation. The average molecular length and quantity of amplifications were validated using the Agilent bioanalyzer instrument (Agilent, United States) and real-time quantitative PCR (EvaGreen™). The final validated libraries were loaded on Illumina Novaseq 6000 platform (Illumina, San Diego, CA, United States) for high-throughput sequencing using a paired-end configuration.

### ITS Genes Amplification and Sequencing

Both *Cryptosporidium*-positive (YangS group) and *Cryptosporidium*-negative (YinC group) feces were used to perform ITS genes amplification. The ITS1 variable region of the fungal ITS gene was amplified using forward primers containing the sequence 5′-CTTGGTCATTTAGAGGAAGTAA-3′ and reverse primers containing the sequence 5′-GCTGCGTTCTTCATCGATGC-3′. Both the primers were tagged with an Illumina adapter for PCR amplification. The annealing temperature was 58°C during 35 PCR cycles. A total of 30 ng verified gDNA was used to perform PCR per reaction. The conditions of purifying PCR products and preparing sequencing libraries were identical with 18S rDNA high-throughput sequencing. Ultimately, paired-end sequencing was performed on qualified libraries using the Illumina Novaseq platform.

### Bioinformatics and Statistical Analysis

Performed the following pre-processed steps on the raw FASTQ format sequences obtained by high-throughput sequencing to eliminate the low quality and adapter pollution for acquiring more accurate and reliable Clean Reads: (1) Quality filtering: those reads (<200 bp) and low quality were filtered using Trimmomatic v0.33 software; (2) To obtain Clean Sequences that do not contain primer sequences, the primer sequences were identified and removed using Cutadapt 1.9.1 software; (3) Both the ends of Clean Reads were stitched through overlap using Usearch v10 software; (4) The UCHIME v4.2 software was used to identify and remove chimera sequences to obtain the final Effective Reads. The obtained high-quality Effective Reads were clustered into operational taxonomic units (OTUs) against the SILVA 132 (18S rDNA high-throughput sequencing) or UNITE (ITS genes high-throughput sequencing) databases at over 97% sequence similarity. A representative sequence per OTU has executed classification and phylogenetic analysis. The Rarefaction, Shannon, and Species accumulation curves evaluated the quality and depth of current sequencing, illustrated by R (v3.0.3) software. Alpha-diversity indexes contained the Chao1 and ACE (calculates species richness), Shannon and Simpson indexes (calculates species diversity), which were processed and calculated by QIIME2 software (v1.7.0). Beta diversity was performed by principal coordinates analysis (PCoA) and combination of (unweighted pair-group method with arithmetic mean) UPGMA clustering tree and histogram, which reflected the difference and similarity of gut fungal community structure in positive and negative infection groups. Metastatic analysis was utilized to assess the fungal community difference between groups. GraphPad Prism (v7.0) and SPSS software (v17.0) were applied to statistical analysis. The values were expressed as the mean ± SD, with *P*-value < 0.05 identified as statistically significant.

## Results

### *Cryptosporidium* Spp. Prevalence in Horses

**FIGURE 1:**
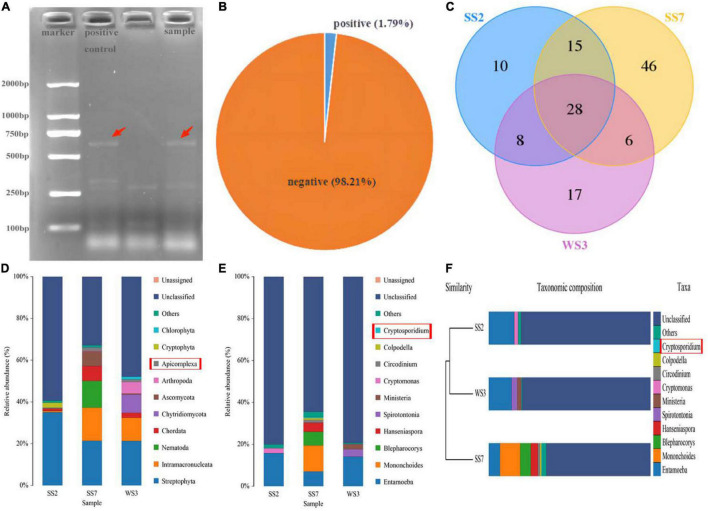
The confirmed positive samples using nested PCR and 18S rDNA high-throughput sequencing. **(A)** Agarose gel electrophoresis. The target bnands are pointed by the red arrows. The first well is a positive control, while the other is a test sample; **(B)** the prevalence of natural infection of *Cryptosporidium* spp. in horses; **(C)** unique and core OTUs of positive samples; **(D,E)** double confirmation of positive samples through 18S high-throughput sequencing; **(F)** cryptosporidium is belonging to the top 10 abundant genus in UPGMA clustering tree.

The positive samples of *Cryptosporidium* infection were preliminarily identified by nested PCR amplification of 18S rDNA genes. Positive samples exhibited a target band of about 587 bp on the agarose gel after staining with ethidium bromide (Figure 1A). The 18S rDNA PCR analysis of 168 rectal fecal samples showed the occurrence of natural *Cryptosporidium* infection in horses of Wuhan, China, to be 1.79% found in three samples (Figure 1B).

### Analysis of 18S rDNA High-Throughput Sequencing Data

The gDNA of feces initially identified as positive for infection was further performed 18S rDNA high-throughput sequencing to confirm *Cryptosporidium* spp. infection. After optimizing the original data, 236,026 effective reads were acquired, and 78,911, 77,938, and 79,177 high-quality valid sequences in the WS3, SS2, and SS7 samples, respectively (Table 1). Based on 97% similarity, 59, 61, and 95 OTUs were separately acquired from WS3, SS2, and SS7, respectively (Figure 1C). Figures 1D–F showed the taxonomic information of the top 10 abundant species identified at the phylum and genus levels. Results showed phylum Apicomplexa and genus *Cryptosporidium*, confirming natural *Cryptosporidium* infection in horses.

**TABLE 1:** The data of 18S rDNA high-throughput sequencing.

Sample ID	Raw reads	Clean reads	Effective reads	Effective (%)
WS3	79,869	79,596	78,911	98.8
SS2	79,954	79,679	77,938	97.48
SS7	79,935	79,624	79,177	99.05

### ITS High-Throughput Sequencing Data Analysis

**FIGURE 2:**
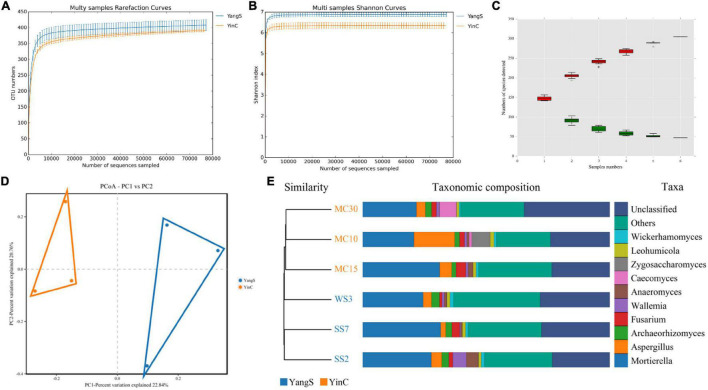
Analysis of samples feasibility and fungal OTUs structures in different groups. **(A)** Rarefaction curves; **(B)** shannon curves; **(C)** species accumulation curves; **(D,E)** fungal PCoA scatterplot and UPGMA clustering tree to reflect similarity between the individuals or groups.

**FIGURE 3:**
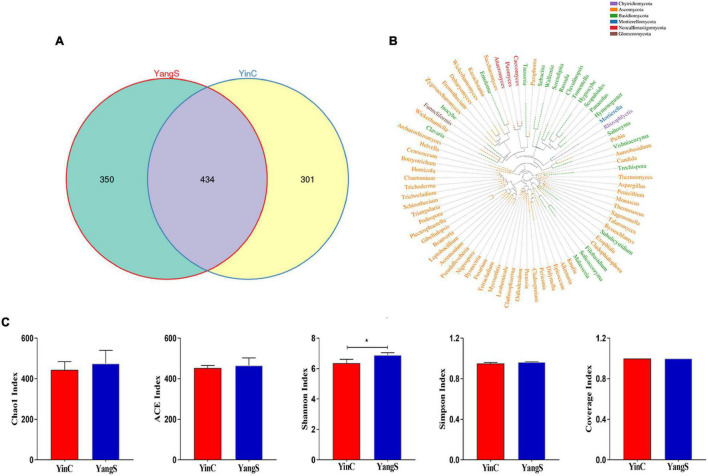
Fungal microbial diversity index and OTUs analysis. **(A)** Unique and core OTUs of fungal community; **(B)** phylogenetic tree at genus level of fungal community; **(C)** alpha diversity indexes.

The ITS amplicon sequence was performed to investigate the alterations in gut fungal community driven by the natural *Cryptosporidium* spp. infection. After the quality control, a total of 478,486 clean reads were obtained, with an average value of 79,748 counts per sample (Table 2). The rarefaction curve, Shannon curve, and species accumulation curve were wide and tended to be flat, suggesting that the current sequencing quantity and depth were reasonable and sufficient to reflect the fungal richness and diversity included in the samples (Figures 2A–C). We recognized a total of 1,519 OTUs based on 97% sequence similarity. Among them, 784 OTUs were acquired for group YangS, and 735 OTUs were acquired for group YinC; among them, 434 OTUs were core OTUs, accounting for 55.36% and 59.05% of the total OTUs in group YangS and group YinC, respectively (Figures 3A,B).

**TABLE 2:** The data of ITS genes high-throughput sequencing.

Sample ID	Raw reads	Clean reads	Effective reads	Effective (%)
SS2	79,834	79,618	78,791	98.69
SS7	80,182	79,992	78,801	98.28
MC10	80,083	79,887	79,046	98.71
MC15	79,673	79,453	78,475	98.5
WS3	80,042	79,799	79,150	98.89
MC30	79,978	79,737	78,495	98.15

### Shifts in Gut Fungal Community Diversity and Structure With the Infection of *Cryptosporidium* Spp.

The Chao1 and ACE indices showed that the difference in species richness between YangS and YinC groups was insignificant (Figure 3C). A significantly lower Shannon diversity index was found in the YinC group than in the YangS group (*p* < 0.05), indicating that the fungal community in the YangS group had higher diversity than the YinC group. However, the other diversity index, Simpson index had no significant difference between these two groups (*p* > 0.05). In addition, the values of coverage index estimates showed excellent coverage in both the groups, ranging from 99.97 to 99.98%. The PCoA based on the fungal OTUs showed that all the fecal samples from the YangS group were clustered together and separated from the YinC group and indicated the variation in the gut fungal community with natural *Cryptosporidium* spp. infection (Figure 2D) was supported by visualizing UPGMA clustering tree associations with horses with or without infected *Cryptosporidium* spp. (Figure 2E).

**FIGURE 4:**
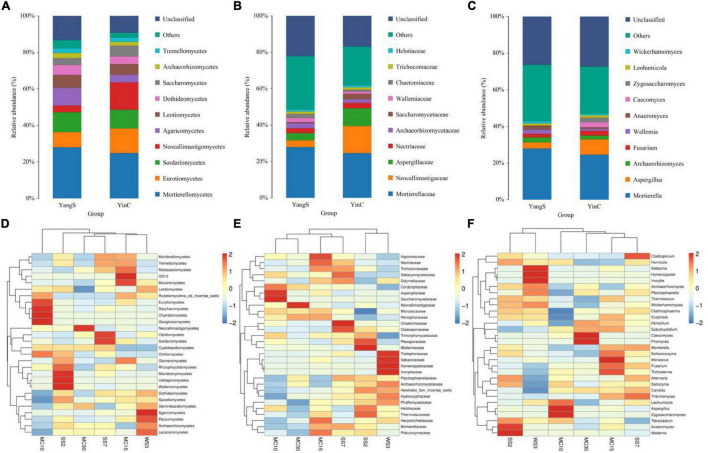
The composition of fungal community in different groups. **(A)** Top 10 abundant taxa at class level; **(B)** top 10 abundant taxa at family level; **(C)** top 10 abundant taxa at genus level; **(D)** heatmap of species abundance clustering at class level; **(E)** heatmap of species abundance clustering at family level; **(F)** heatmap of species abundance clustering at genus level.

Mapping different taxonomical levels (class, family, and genus levels) relative abundances onto the species distribution histogram revealed gut fungal community structure in horses with or without infected *Cryptosporidium* spp. (Figures 4A–C). Significantly, the same species hosts generally had similar phylum-level abundances, e.g., phyla Mortierellomycota and Ascomycota were dominant in fungal communities regardless of *Cryptosporidium* spp. infection, which consisted of over 25% of total tags on average. Besides these two dominant phyla, the abundantly present phylum was Basidiomycota (16.1%) in the YangS group, while Neocallimastigomycota (15%) was observed as the predominant phylum in YinC group. Classes Eurotiomycetes (8.44, 13.40%), Mortierellomycetes (28, 24.9%), and Sordariomycetes (10.8, 10.3%) were the most abundant in both YangS and YinC groups, whereas Agaricomycetes (9.6%) and Neocallimastigomycetes (15%) were enriched in the YangS and YinC groups, respectively. The outcomes of the taxonomic classification level of the gut fungal community identified one dominating family at its level, Mortierellaceae, accounting for 24.6% of identified taxonomic sequences. Besides, Aspergillaceae (3.99%) and Neocallimastigaceae (15%) dominated the YangS and YinC groups, respectively. Using genus-level cluster analysis, *Mortierella* was the predominant genus of total sequences in both groups, consisting of over 24.6% of total tags, whereas *Aspergillus* was secondary (over 3.24%). Moreover, the most abundant genera in the YangS group were *Archaeorhizomyces* (2.82%), *Wallemia* (2.29%), *Anaeromyces* (2.14%), *Fusarium* (1.92%), and *Inocybe* (1.33%). By comparison, *Caecomyces* (2.67%), *Fusarium* (2.62%), *Zygosaccharomyces* (2.53%), *Archaeorhizomyces* (2.06%), and *Piromyces* (1.37%) were enriched in the YinC group. Furthermore, the heatmap results exhibited relative species abundance at different levels (class, family, and genus) were further validated by analytical strategy (Figures 4D–F).

### Changes in Gut Fungal Composition of Horses With the Infection of *Cryptosporidium* Spp.

**FIGURE 5:**
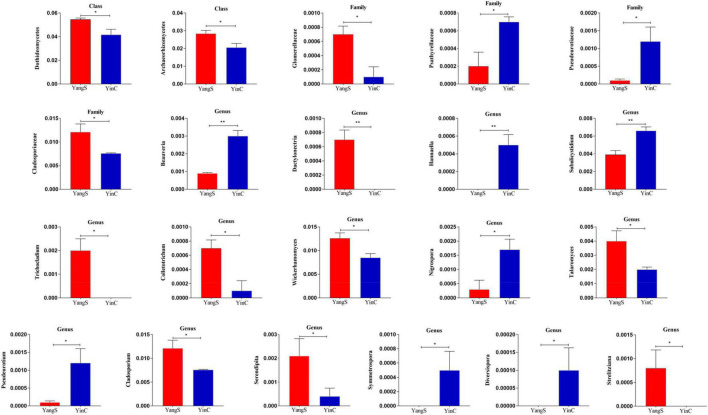
Significant differences in fungal microbiota abundance. *p < 0.05; **p < 0.01.

Metastatic analysis was used to explore the significant changes in the gut fungal community at class, family, and genus levels to undertake exploratory shifts in the gut fungal microbiota of natural *Cryptosporidium* spp. infection (Figure 5). At the class level, 2 taxa were significantly different when comparing YangS and YinC in the exploration panel (*p* < 0.05), which were Dothideomycetes and Archaeorhizomycetes. In comparing all the taxa between YangS and YinC, four families had different abundances in the combined panel; families Glomerellaceae and Cladosporiaceae were enriched in YangS, while families Psathyrellaceae and Pseudeurotiaceae were enriched in YinC (all: *p* < 0.05). Results of the genus-level cluster showed that 304 genera were recognized across all the sequences. Several taxa at the genus level were found to have significant infection-related variations. The high signals of the YangS group of fungi were enriched at these genera levels (*Dactylonectria*, *Trichocladium*, *Colletotrichum*, *Wickerhamomyces*, *Talaromyces*, *Cladosporium*, *Serendipita*, and *Strelitziana*). Conversely, *Beauveria*, *Hannaella*, *Subulicystidium*, *Nigrospora*, *Pseudeurotium*, *Symmetrospora*, and *Diversispora* were represented significantly in YinC group compared with YangS group (*p* < 0.05 or *p* < 0.01).

## Discussion

Microbiota typically exceeds intestinal parasites in biomass, while commensal fungi and parasites occupy different taxonomic spaces; both have responded to evolutionary capability by developing some strategies for modulating host immunity (Sender et al., 2016). It is evident that they have established dialog with a common theme of creating new homeostasis in the host gut. Specifically, parasitic infection changes the structure and composition of the gut microbiota, and conversely, the inherent microbiota affects invasions of new parasite species within hosts (Berrilli et al., 2012). The bacterial microbiota patterns associated with susceptibility to *Cryptosporidium* infection have been identified in humans (Chappell et al., 2016), while the theme of the fungal–parasite interaction was relatively unexplored. In the current study, we first performed an epidemiological survey to investigate the condition of natural *Cryptosporidium* infection in horses of Wuhan, P. R. China. By doing so, we obtained the positively infected feces to investigate the gut microbiome in which fungal communities have been changed by exposure to *Cryptosporidium* spp.

*Cryptosporidium* spp. are protozoan parasites of medical importance that causes moderate-to-severe diarrhea and death in varieties of vertebrate hosts (Abdoli and Maspi, 2018). *Cryptosporidium* spp. are now considered important waterborne parasites worldwide. The positively infected horses pose a serious public health threat in many countries, for example, the United States and France (Coleman et al., 1989; Ma et al., 2019). The main symptoms includes diarrhea, abdominal pain, vomiting and low-grade fever (Current and Garcia, 1991). In 1976, the first case of human cryptosporidiosis was reported in patient with severe watery diarrhea (Nime et al., 1976). The severity of *Cryptosporidium* infection depends usually on the host factors e.g., immunocompetent individuals suffer a transient self-limiting illness (Hunter and Nichols, 2002). When *Cryptosporidium* infection was considered as a major inducement of diarrhea in immunocompetent individuals, the public health meaning of it became apparent (Tzipori et al., 1983). Evidence from epidemiology and microbiology revealed the direct and indirect transmission routes of *Cryptosporidium* spp.: (1) direct infection occurs by the fecal–oral route, including animal-to-human (zoonotic), animal-to-animal, human-to-human, and human-to-animal transmissions (Bouzid et al., 2013); (2) Indirect infection occurs through environmental contamination, involving contact with *Cryptosporidium* fecal contaminated material (Jiang et al., 2005). The infection rate in adult equines ranges from 0.33 to 9% (Sturdee et al., 2003) with 1.79% prevalence of natural *Cryptosporidium* spp. The current study was consistent with previous reports in Algeria (2.3%) and China’s southwest (1.8%) (Jian et al., 2016; Deng et al., 2017). It was inconsistent with previous reports of prevalence in cattle and donkeys (Ma et al., 2015; Li et al., 2019). We inferred that the potential reason might due to the different animal species that had different immunity against *Cryptosporidium* spp. infection.

The composition and structure of the intestinal microbiome can be influenced by diet, age, environmental factors, and the role of protozoal parasites (Yatsunenko et al., 2012; Fan et al., 2014; Mishra et al., 2014; Martinez et al., 2015), but few studies focusing on gut fungi have been performed with *Cryptosporidium* spp. infection. In neonatal mice, the innate responses requiring microbiota played a key role in protection against *Cryptosporidium* infection (Lantier et al., 2014). Although the impacts of microbiome parasite on the host were predictable in the long term, the potential competition or facilitation in the intestinal micro-ecosystem might happen in a much shorter timeframe (McKenney et al., 2015). After 3 days of *Cryptosporidium* infection, as seen in protein-malnourished mice, intestinal injury and inflammation were recorded (Bolick et al., 2017). The composition of gut microflora in monkeys also shifted significantly following *Cryptosporidium* infection (McKenney et al., 2017). According to a previous report, in all conditions with significant alteration, a higher diversity of bacteria seems to associate with infections (Aivelo and Norberg, 2018), which was consistent with the current results. More Shannon diversity index was found in natural *Cryptosporidium* infection horses. Moreover, six branches in the UPGMA evolutionary tree were sequestered, showing the segregation of fungal community structure and composition in *Cryptosporidium* infections. Such findings supported the hypothesis about the response of fungal microbiome to *Cryptosporidium* infection in horses (McKenney et al., 2017).

As mentioned previously, resident microbiota composition could affect the success rate of invading parasites; meanwhile, parasites actively secrete molecules affecting microbiota composition. This information conveyed that the changes in some functional microflora abundance exacerbate infection susceptivity or that particular gut condition, which infected *Cryptosporidium* spp., drives the shifts of the functional microorganisms. The significant enrichment of the classes Dothideomycetes and Archaeorhizomycetes contributed to the unique gut fungal signature found in YangS group in this study. Although research on the fungal microbiota is still in its early stages, various reports have revealed its potential effect on maintaining hosts’ homeostasis. It coexists with bacterial microorganisms and substantially expands gut microecology interacting with the intestinal immune system. Mainly fungal microorganisms were contributed to decomposing the lignocellulose in the gut and implementing this functionality through secretion of cell wall degradation enzymes and physical penetration (Wang et al., 2021a). The final metabolites of this procedure were mainly formate, hydrogen, and acetate (Gruninger et al., 2014). Class Dothideomycetes is the largest taxa in the kingdom of fungi, many of which have evolved to an incredible diversity of lifestyles. The major ecological niche of this class is plant pathogens, and most food crops and feedstocks for biofuel production are its’ infected subjects (Haridas et al., 2020). While the ecology of class Archaeorhizomycetes is poorly resolved, their abundance in the soil is currently disputed. Even so, Tonjer et al. (2021) reported that these root-associated ascomycetes probably promote high Carbon turnover. At the family level, we found one taxa, affiliated with degraded hemicellulose, significantly enriched in the YinC group, i.e., Pseudeurotiaceae (Leung et al., 2016). In contrast, classes Glomerellaceae and Cladosporiaceae had the opposite trend (enrichment in YangS group). Cladosporium species is an opportunistic human pathogen belonging to the class Cladosporiaceae, reported from cases of various superficial and invasive infections worldwide (Batra et al., 2019). Coincidentally, leaf anthracnose is caused by genus Colletotrichum, a notorious necrotrophic fungus that belongs to the family Glomerellaceae (Wang et al., 2021b). These variations in the abundances of involved fungal microflora might be associated with infection development. The robust connection warrants further study.

At the genus level, *Wickerhamomyces*, *Talaromyces*, and *Cladosporium* were recorded as most abundant in positively infected horses (all: *p* < 0.05), probably associating with their physiological effects in the gut. For example, genus *Talaromyces marneffei*, an essential pathogenic thermally dimorphic fungus, have been reported of causing clinical systemic mycosis in patients with human immunodeficiency virus (HIV) or non-HIV infected with damaged cell-mediated immunity (Chan et al., 2016). Although the genus *Wickerhamomyces* has been used in various biotechnology applications of agriculture, some studies have recognized this specie as an opportunistic human pathogen (Nundaeng et al., 2021). Remarkably, several microbial components were found connected to plant pathogen, i.e., *Dactylonectria*, *Colletotrichum*, and *Cladosporium*, in YangS group (Parkinson et al., 2019). There is no knowledge about the physiological effects of plant pathogens in vertebrate intestines. From current data, we speculated that its enrichment in the gut might not be an accidental phenomenon, which is worthy of further advancement in the field of fungi. The substantial rise in taxa connected with health threats in *Cryptosporidium* infected horses was followed by a significant decrease in several beneficial fungi classifications. *Beauveria* was reported for its eminent function as a mycoinsecticide and tool in the integrated pest management (Mascarin and Jaronski, 2016), which might consist with down-regulation of fungi diversity in negative infected horses because its highest abundance was recorded in YinC group (*p* < 0.01). The secondary metabolites produced from a piezotolerant fungus, *Nigrospora* spp., exhibited great antimicrobial and anticancer activities and has been applicated in pharmaceutical industries (Arumugam et al., 2015), while *Diversispora* as an arbuscular mycorrhizal symbiosis, can stimulate growth and nutrient uptake in host plants (Chen et al., 2017). *Cryptosporidium* spp. survival in the hosts’ gut must interact with intestinal constituents to compensate for deficiencies, i.e., lack of numerous metabolic systems (Alcock et al., 2012). Gut microbiome–parasite relationship was demonstrated to be a medium in modulating significant biochemical changes during cryptosporidiosis (Karpe et al., 2021). Charania et al. (2020) identified that specific changes in the gut microbiome allow for more favorable survival of *Cryptosporidium* parasite in mice (Charania et al., 2020), hence this conclusion supported the current results. A question, which could only be speculated upon, is whether changes in the abundance of fungal microflora is inducer in *Cryptosporidium* infection; do the microflora changes represent an actual connection between the intestinal conditions and *Cryptosporidium* infection, or are they indirectly involved in infection? Anyway, it was speculated that the marked fungal microbiota alterations in YangS were involved in infection.

## Conclusion

The present research focused on the prevalence of *Cryptosporidium* spp. infection in horses. According to our findings, the positive samples exhibited an increased abundance of fungi associated with health issues, while taxa associated with beneficial physiological effects on the host showed the opposite effect. Consequently, our findings suggest that certain modifications in the host fungal microbiome might enable *Cryptosporidium* parasites to survive. These findings might pave the way for creating a therapeutic or preventative approach that explicitly targets fungal microorganisms that produce infection susceptibility or resistance.

## Data Availability Statement

The raw data of current study had been submitted to NCBI Sequence Read Archive (SRA) with accession no: PRJNA797580.

## Ethics Statement

The animal study was reviewed and approved by Ethics Committee of Huazhong Agricultural University and Animals Research Centre of Hubei province.

## Author Contributions

YaW, KL, and YiW: research idea and methodology. XL, KD, HL, and XC: reagents, materials, and analysis tools. YaW: writing – original draft and preparation. MK and ZB: writing – review and editing. KL: visualization and supervision. All authors known and approved the final manuscript.

## Conflict of Interest

KD was employed by China Tobacco Henan Industrial Co., Ltd., China. The remaining authors declare that the research was conducted in the absence of any commercial or financial relationships that could be construed as a potential conflict of interest.

## Publisher’s Note

All claims expressed in this article are solely those of the authors and do not necessarily represent those of their affiliated organizations, or those of the publisher, the editors and the reviewers. Any product that may be evaluated in this article, or claim that may be made by its manufacturer, is not guaranteed or endorsed by the publisher.
